# Supplementary material for: Novel Driver Strength Index highlights important cancer genes in TCGA PanCanAtlas patients
Source: PeerJ. 2022 Aug 11;10:e13860. doi: 10.7717/peerj.13860 (PMC9375969; doi:10.7717/peerj.13860)
Supplement: Supplemental Information 2 [file peerj-10-13860-s002.zip › COHORTS/genes plots/detailed/2021_8_16_14_9_distribution_events_detailed_males_37.pdf]

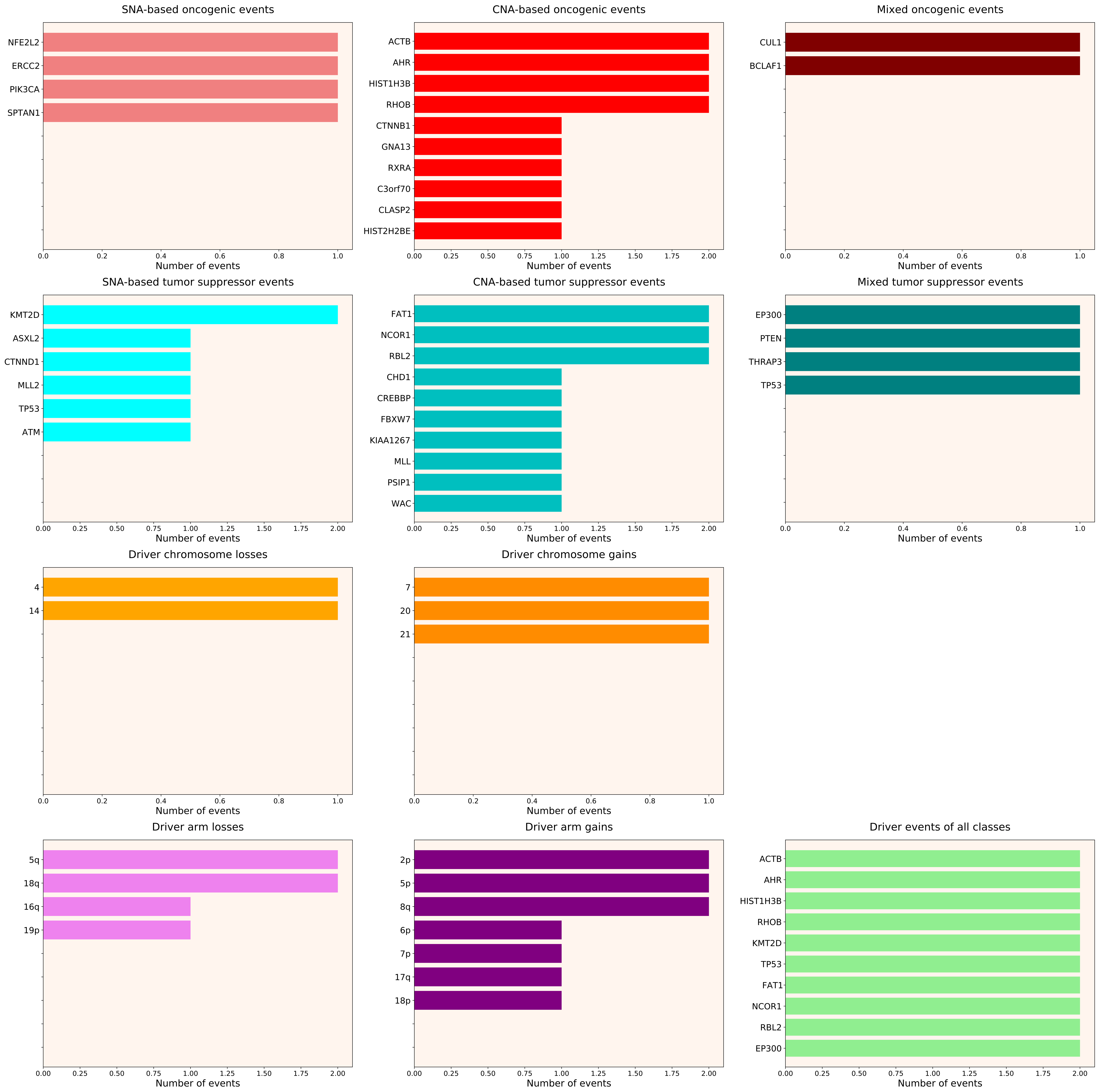
SNA-based oncogenic events
NFE2L2
ERCC2
PIK3CA
SPTAN1
0.0 0.2 0.4 0.6 0.8 1.0
Number of events
CNA-based oncogenic events
ACTB
AHR
HIST1H3B
RHOB
CTNNB1
GNA13
RXRA
C3orf70
CLASP2
HIST2H2BE
0.00 0.25 0.50 0.75 1.00 1.25 1.50 1.75 2.00
Number of events
Mixed oncogenic events
CUL1
BCLAF1
0.0 0.2 0.4 0.6 0.8 1.0
Number of events
SNA-based tumor suppressor events
KMT2D
ASXL2
CTNND1
MLL2
TP53
ATM
0.00 0.25 0.50 0.75 1.00 1.25 1.50 1.75 2.00
Number of events
CNA-based tumor suppressor events
FAT1
NCOR1
RBL2
CHD1
CREBBP
FBXW7
KIAA1267
MLL
PSIP1
WAC
0.00 0.25 0.50 0.75 1.00 1.25 1.50 1.75 2.00
Number of events
Mixed tumor suppressor events
EP300
PTEN
THRAP3
TP53
0.0 0.2 0.4 0.6 0.8 1.0
Number of events
Driver chromosome losses
4
14
0.0 0.2 0.4 0.6 0.8 1.0
Number of events
Driver chromosome gains
7
20
21
0.0 0.2 0.4 0.6 0.8 1.0
Number of events
Driver arm losses
5q
18q
16q
19p
0.00 0.25 0.50 0.75 1.00 1.25 1.50 1.75 2.00
Number of events
Driver arm gains
2p
5p
8q
6p
7p
17q
18p
0.00 0.25 0.50 0.75 1.00 1.25 1.50 1.75 2.00
Number of events
Driver events of all classes
ACTB
AHR
HIST1H3B
RHOB
KMT2D
TP53
FAT1
NCOR1
RBL2
EP300
0.00 0.25 0.50 0.75 1.00 1.25 1.50 1.75 2.00
Number of events
